# Evaluation of iron overload by cardiac and liver T2* in β-thalassemia: Correlation with serum ferritin, heart function and liver enzymes

**DOI:** 10.34172/jcvtr.2021.18

**Published:** 2021-02-18

**Authors:** Hengameh Khadivi Heris, Babak Nejati, Khatereh Rezazadeh, Hossein Sate, Roya Dolatkhah, Zohreh Ghoreishi, Ali Esfahani

**Affiliations:** ^1^Department of Internal Medicine, School of Medicine, Tabriz University of Medical Sciences, Tabriz, Iran; ^2^Hematology and Oncology Research Center, Tabriz University of Medical Sciences, Tabriz, Iran; ^3^Nutrition Research Center, School of Nutrition & Food Science, Tabriz University of Medical Sciences, Tabriz, Iran; ^4^Department of Cardiology, School of Medicine, Tabriz University of Medical Sciences, Tabriz, Iran; ^5^Nutrition Research Center, Department of Clinical Nutrition, School of Nutrition & Food Science, Tabriz University of Medical Sciences, Tabriz, Iran

**Keywords:** Cardiomyopathy, T2* Magnetic Resonance Imaging, Ferritin, Iron Overload, β-Thalassemia

## Abstract

***Introduction:*** In this study, we aimed to assess the relationship of cardiac and hepatic T2* magnetic resonance imaging (MRI) values as a gold standard for detecting iron overload with serum ferritin level, heart function, and liver enzymes as alternative diagnostic methods.

***Methods:*** A total 58 patients with beta-thalassemia major who were all transfusion dependent were evaluated for the study. T2* MRI of heart and liver, echocardiography, serum ferritin level, and liver enzymes measurement were performed. The relationship between T2* MRI findings and other assessments were examined. Cardiac and hepatic T2* findings were categorized as normal, mild, moderate, and severe iron overload.

***Results:*** 22% and 11% of the patients were suffering from severe iron overload in heart and liver, respectively. The echocardiographic findings were not significantly different among different iron load categories in heart or liver. ALT level was significantly higher in patient with severe iron overload than those with normal iron load in heart (*P* =0.005). Also, AST level was significantly lower in normal iron load group than mild, moderate, and severe iron load groups in liver (*P* <0.05). The serum ferritin level was significantly inversely correlated with cardiac T2* values (r = -0.34, *P* =0.035) and hepatic T2* values (r = -0.52, *P* =0.001).

***Conclusion:*** Cardiac and hepatic T2* MRI indicated significant correlation with serum ferritin level.

## Introduction

β-thalassemia is a hereditary disease resulting from defective hemoglobin production that causes chronic and severe hemolytic anemia.^1^ Patients with β-thalassemia who need multiple blood transfusions are prone to develop iron overload as a consequence of inadequate chelation therapy, erythrocyte catabolism, hypertransfusion, and excessive iron absorption from the gastrointestinal tract. The heart, liver and various endocrine glands are the major sites for iron deposition that iron overload in these organs induces serious damage to them.^2^

Iron-induced cardiomyopathy is considered as the most prevalent cause of mortality in patients with thalassemia major which could be reversed if effective chelation therapy is initiated on time.^3,4^ Moreover, iron storage in hepatocytes, major deposition site for body iron, may develop liver disease as evidenced by increased liver enzymes activity including alkaline phosphatase (ALP), alanine transaminase (ALT), and aspartate aminotransferase (AST),^5,6^ Therefore, early diagnosis of iron overload in the heart and liver would allow to use appropriate iron-chelating therapy to improve morbidity and increase survival of patients.^7^

Previous studies reported several methods to evaluate iron burden in patients with thalassemia including total iron binding capacity (TIBC), serum iron, serum ferritin, liver biopsy, echocardiography, and T2^*^ magnetic resonance imaging (MRI).^8,9^ Some studies indicated that ferritin level is well correlated with iron accumulated in the organs,^10,11^ but its level may be influenced by inflammatory conditions, infectious, and malignant disease.^12^ MRI using gradient echo T2^*^ has been identified as a gold standard non-invasive technique to quantify tissue iron levels.^13,14^ Iron deposited in heart is correlated with cardiac function as assessed by echocardiologhy.^15^ Therefore, we aimed to evaluate the cardiac and liver iron deposited using MRI T2^*^ in patients with beta-thalassemia, and to study correlation between MRI T2^*^ outcomes with serum ferritin level, liver enzymes activity and also cardiac function measured by echocardiography.

## Materials and Methods

### Subjects

This cross-sectional study was carried out on 58 patients with β-thalassemia major who were all transfusion dependent in Shahid Ghazi Tabatabaei Hospital, Tabriz, Iran over November 2011 to October 2016. 43% of patients received desferal, 11% received deferiprone, and 46% received combination of desferal and deferiprone. The patients suffering from β-thalassemia major who were older than 15 years old of age and were transfusion dependent were included in the study. Those patients with valvular heart disease, known history of heart failure, major congenital heart disease and infectious disease were excluded. The patients were also excluded if had not attended regularly and had not consumed their therapy.

### Biochemical measurement

The activity of ALT, AST, and AL enzymes was evaluated using colorimetric method by commercial kits (Randox, Crumlin, UK). Hemoglobin level were also measured by cyanmethemoglobin method.

### Magnetic resonance imaging

Cardiac and liver MRI T2^*^ assessment was performed using 1.5 Tesla MRI device (Magnetom Simphony; Siemens, Erlangen, Germany) to assess the quantity of iron deposition in heart and liver tissue. The T2^*^ MRI values were calculated by “CMR Tools” software (London, UK).^16^ The patients were classified as normal myocardium if cardiac T2^*^ MRI values were > 20 ms, mild heart iron overload if they were 15-20 ms, moderate heart iron overload if they were 10-15 ms, and severe heart iron overload if they were < 10 ms.^17^ Cardiac MRI T2* was performed for 46 patients (31 males, 15 females). Based on hepatic T2* image values, the level of iron overload severity in liver were reported in four classes: normal (T2^*^ > 6.3 ms), mild (T2^*^ 2.8-6.3 ms), moderate (T2^*^ 1.4-2.8 ms), and severe (T2^*^ <1.4 ms).^18^ Forty-five patients (30 males, 15 females) underwent hepatic MRI T2* examination.

### Serum ferritin level

Serum ferritin level was measured by human ELISA kit (Thermo Fisher, Vilnius, Lithuania). The color changes were detected at wavelength 450 nm using an ELISA microplate reader 2100 Stat Fax (Awareness Technology. Inc., USA) based on the instructions provided by the manufacturer.

### Echocardiography

Two-dimension (2D), M mode, color Doppler and Tissue Doppler Imaging (TDI) echocardiography was performed using Vivid 7 Dimension (GE Healthcare, USA) with a 2.5 or 3.5 MHz phased array transducer. In all patients, echocardiography was performed after receiving the packed cell and correcting the hemoglobin level. The measurement of the left ventricular diastolic function (LVDF), the left ventricular systolic function (LVSF), the right ventricular systolic function (RVSF), and the pulmonary arterial pressure (PAP) was conducted according to the recommendation of the American Society of Echocardiography (ASE).^19^ Echocardiographic assessment was performed by an expert cardiologist who was not aware of T2^*^ values and serum ferritin level on 53 patients with β-thalassemia major (37 males, 16 females).

### Statistical analysis

Data were analyzed using SPSS software version 16 (SPSS Inc., USA). The data distribution was assessed visually and using the Kolmogorov–Smirnov goodness of fit. The results were reported as number (percentage) for categorical variables, as mean ± standard deviation (SD) for normally distributed variables, and as median (interquartile range (IQR)) for non-normally distributed variables. To assess the correlation of liver and cardiac T2^*^ with serum ferritin level the Spearman’s tests were used. Moreover, we used one-way analysis of variance (ANOVA) and Kruskal-Wallis test to compare the serum ferritin, cardiac function, liver enzymes, and hemoglobin level between the cardiac or liver iron load categories for normal and non-normal data, respectively. Post hoc paired comparisons were performed by a Mann-Whitney *U* test for normal distributed data and by a Sidak test for non-normal distributed data. Categorical data were compared using the chi-square and Fisher exact test. We used the independent-sample t-test to assess the differences in HB level between two categories of LVSF (preserved, reduced), RVSF (preserved, reduced) and PAP (preserved, increased). A *P* < .05 was as the threshold value of statistical significance.

## Results

### Characteristics of patients

We studied 58 patients with β-thalassemia major who were transfusion dependent. The general characteristics of the patients are shown in Table 1. Forty patients (69.0%) were male and the median (IQR) of the patients age was 22 y (20 to 25 year). According to the echocardiography results, cardiac function was normal in the most of the patients; 46 patients (87%) revealed preserved LVSF, 51 patients (96%) indicated preserved RVSF, 52 patients (98%) had normal LVDF, and 41 patients (77%) showed normal PAP. Moreover, the MRI T2^*^ results indicated that the median (IQR) of iron overload in heart and liver was 16.9 ms (10.2 to 25.3 ms) and 2.4 ms (1.8 to 4.3 ms), respectively. Ten (22%) patients were assigned to the severe iron overload in myocardium tissues and 5 (11%) patients were assigned to the severe iron overload in hepatic tissues.

**Table 1:** General characteristics of the study patients^a^

**Variables**	**Values**
Age, median (IQR), years	22 (20-25)
Gender, n (%) Male, n (%) Female, n (%)	58 (100) 40 (69) 18 (31)
Diabetes mellitus, n (%)	11 (25.6)
Ferritin, median (IQR), ng/mL	3460 (1876-5757)
LVSF ,n(%) Preserved Mildly reduced Moderately and severely reduced	46 (87) 4 (7) 3 (6)
RVSF, n (%) Preserved Mildly reduced Moderately and severely reduced	51 (96) 1 (2) 1 (2)
LVDF, n (%) Preserved Moderately and severely reduced	52 (98) 1 (2)
Cardiac iron load, n (%) Normal Mild overload Moderate overload Severe overload	46 (100) 20 (43.5) 5 (10.9) 11 (23.9) 10 (21.7)
PAP, n (%) Normal Mildly increased Moderately and severely increased	41 (77) 10 (19) 2 (4)
Hepatic iron load, n (%) Normal Mild overload Moderate overload Severe overload	45 (100) 10 (22.2) 10 (22.2) 20 (44.4) 5 (11.1)
AST, median (IQR), u/L	37 (23-50.5)
ALT, median (IQR), u/L	40 (19.5-68)
ALP, median (IQR), u/L	321 (198-490)
Bilirubin total, median (IQR), mg/dL	2.2 (1.64-3.71)
Bilirubin direct, median (IQR), mg/dL	0.30 (0.22-0.43)

Abbreviations: ALP, alkaline phosphatase; ALT, alanine transaminase; AST, aspartate aminotransferase; IQR, interquartile range; LVDF, left ventricular diastolic function; LVSF, left ventricular systolic function; RVSF, right ventricular systolic function; PAP, pulmonary arterial pressure; ^a^This study was carried out on 58 patients, however, echocardiography was performed on 53 patients and cardiac MRI was done on 46 patients and hepatic MRI was performed on 45 patients

The HB level of patients was not different between two categories of LVSF and RVSF and PAP (data was not shown).

### Cardiac MRI T2*

The patients were divided to four categories of iron load in heart based on myocardium T2^*^ results as normal, mild, moderate, and severe iron load. The mean MRI T2^*^ times was significantly different among four groups. As shown in Table 2, there were not significant differences among four iron overload groups regarding sex, diabetes mellitus, serum ferritin level, ejection fraction (EF) cardiac function parameters (LVSF, RVSF, PAP), AST level, ALP level, total bilirubin, direct bilirubin, and hemoglobin level. However, the ALT level was significantly different among groups. Post Hoc Mann-Whitney U test paired comparisons indicated that patient with severe iron load had significantly higher ALT level than patients with normal iron load (*P* = 0.005).

**Table 2:** Comparison of sex, having diabetes mellitus, cardiac function and liver enzymes among four groups of patients with β-thalassemia with different levels of iron deposition according to cardiac T2^*^ MRI

	**Cardiac iron load**				
**Variables**	**Normal**	**Mild**	**Moderate**	**Severe**	***P***
T2^*^, mean ± SD,ms	27.7 ± 5.5	17.5 ± 1.8	12.6 ± 1.2	7.9 ± 1.2	<0.001^*^
Gender n (%) Male Female	12 (60.0) 8 (40.0)	4 (80.0) 1 (20.0)	9 (81.8) 2 (18.2)	6 (60.0) 4 (40.0)	0.578^#^
Diabetes mellitus, n (%) Yes No	3 (16.7) 15 (83.3)	2 (40) 3 (60.0)	5 (50.0) 5 (50.0)	1 (10.0) 9 (90.0)	0.132^#^
Ferritin, median (IQR), ng/mL	2440.0 (1747.0-4150.0)	4570.0 (2378.5-5514.5)	2554.0 (1828.0-9500.0)	5134.0 (2862.7-8942.5)	0.060^δ^
EF (%)	55.0 (55.0-60.0)	55.0 (27.5-57.5)	57.5 (53.7-60.0)	55.0 (55.0-60.0)	0.527
LVSF n (%) Preserved Reduced	15 (88.2) 2 (11.8)	3 (75.0) 1 (25.0)	8 (72.7) 3 (27.3)	10 (100.0) 0	0.252^#^
RVSF n (%) Preserved Reduced	17 (100.0) 0	3 (75.0) 1 (25.0)	11 (100.0) 0	10 (100.0) 0	0.095^#^
PAP n (%) Preserved Increased	11 (64.7) 6 (35.3)	3 (75.0) 1 (25.0)	9 (81.8) 2 (18.2)	10 (100.0) 0	0.181^#^
AST, median (IQR), u/L	29.0 (18-37)	42.5 (39.2-65.2)	40.0 (21.7-56)	45.0 (34.5-77.5)	0.068^δ^
ALT, median (IQR), u/L	21.0 (18.0-51.0)	53.0 (42.7-64)	35.0 (21.5-62.2)	60 (54.5-90)	0.024^δ^
ALP, median (IQR), u/L	277.0 (247.0-519.0)	343.0 (156.0-650)	345.0 (286.0-581.0)	227.0 (155.7-322.5)	0.142^δ^
Bilirubin total, median (IQR), mg/dL	2.3 (1.5-3.1)	5.7 (1.2-5.7)	2.0 (1.6-3.4)	2.1 (1.8-2.7)	0.837^δ^
Bilirubin direct, median (IQR), mg/dL	0.3 (0.2-0.5)	0.3 (0.2-0.3)	0.3 (0.2-0.4)	0.3 (0.2-0.3)	0.778^δ^
Hemoglobin, median (IQR), mg/dL	9.4 (9.0-10.0)	10.0 (9.2-10.7)	9.9 (8.3-11.0)	9.1 (8.6-9.7)	0.408^δ^

Abbreviations: ALP, alkaline phosphatase; ALT, alanine transaminase; AST, aspartate aminotransferase; EF: ejection fraction; IQR, interquartile range; LVSF: left ventricular systolic function; PAP, pulmonary arterial pressure; RVSF: right ventricular systolic function; SD, standard deviation *ANOVA test ^#^Fisher exact test ^δ^Kruskal-Wallis test

**Figure 1:**
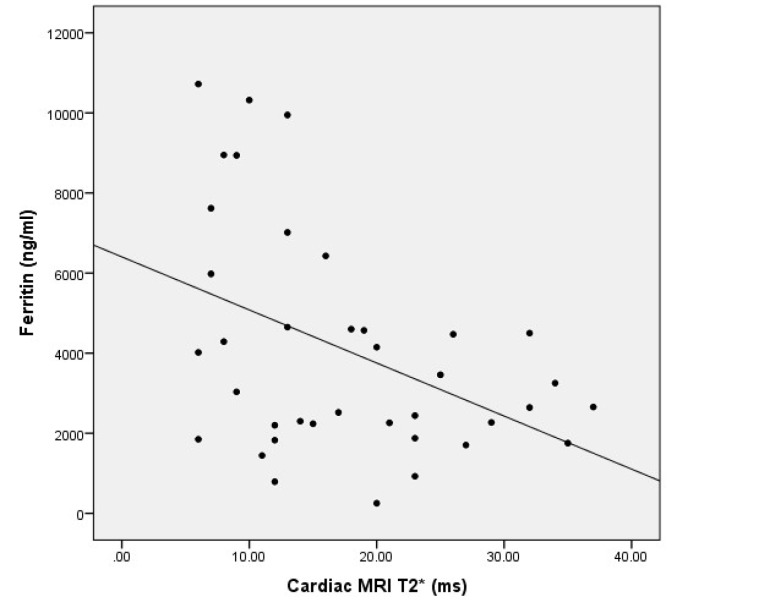
Correlation between cardiac MRI T2^*^ times and serum ferritin level in β-thalassemia patients (r = -0.34, *P* = 0.035)

Furthermore, there was a significant inverse linear correlation between serum ferritin level and heart MRI T2^*^ (r = -0.34, *P* = 0.035) (Figure 1).

### Hepatic MRI T2*

Based on the results of liver T2^*^, patients were categorized to four groups including normal, mild, moderate, and severe iron load. In Table 3, the results of comparison of sex, having diabetes mellitus, cardiac function and liver enzymes among four groups of patients with β-thalassemia with different levels of iron overload according to hepatic T2^*^ MRI were shown. The hepatic T2^*^ times showed significantly different values among four groups of patients. The patients in four categories of iron deposition in liver did not show significantly different sex, having diabetes mellitus, EF, cardiac function (LVSF, RVSF, PAP), ALT level, ALP level, total bilirubin, direct bilirubin, and hemoglobin level. However, serum ferritin level was significantly different among groups of patients. Post Hoc Sidak test paired comparisons showed that in patient with normal iron load, serum ferritin level was significantly lower than patients with moderate and severe iron load, as well as, patients with mild iron load had significantly lower serum ferritin than patient with moderate and severe iron load. Moreover, there was significantly different AST level among patients in groups, which in normal iron load group, AST level was significantly lower than mild, moderate and severe iron load groups according to the post Hoc Mann-Whitney U test paired comparisons.

**Table 3:** Comparison of sex, having diabetes mellitus, cardiac function and liver enzymes among four groups of patients with β-thalassemia with different levels of iron deposition according to hepatic T2^*^ MRI

	**Hepatic iron load**				
**Variables**	**Normal**	**Mild**	**Moderate**	**Severe**	***P***
T2^*^ median (IQR), ms	8.7 (7.1-15.4)	3.4 (3.2-5.2)	2.0 (1.6-2.4)	1.3 (1.0-1.3)	<0.001^δ^
Gender, n (%) Male Female	5 (50.0) 5 (50.0)	8 (80.0) 2 (20.0)	12 (60.0) 8 (40.0)	5 (100.0) 0	0.177^#^
Diabetes mellitus, n (%) Yes No	1 (11.1) 8 (88.9)	2 (25.0) 6 (75.0)	5 (25.0) 15 (75.0)	3 (60.0) 2 (40.0)	0.317^#^
Ferritin, mean ± SD, ng/mL	2327.7 ± 1672.5	2755.1 ±1176.7	5034.5 ± 3028.8	6103.6 ± 3495.4	0.009^*^
EF (%)	55.0 (45.0-60.0)	55.0 (50.0-60.0)	55.0 (55.0-60.0)	55.0 (55.0-60.0)	0.615
LVSF, n (%) Preserved Reduced	8 (80.0) 2 (20.0)	6 (85.7) 1 (14.3)	16 (84.2) 3 (15.8)	5 (100.0) 0	0.925^#^
RVSF, n (%) Preserved Reduced	10 (100.0) 0	7 (100.0) 0	18 (94.7) 1 (5.3)	5 (100.0) 0	1.000^#^
PAP, n (%)] Preserved Increased	6 (60.0) 4 (40.0)	7 (100.0) 0	14 (73.7) 5 (26.3)	5 (100.0) 0	0.169^#^
AST, median (IQR), u/L	21.0 (14.2-29.7)	37.0 (23.0-61.5)	40.5 (28.5-66.5)	40.0 (39.5-70.0)	0.032^δ^
ALT, mean ± SD, u/L	21.6 ± 9.1	49.6 ± 34.9	55.7 ± 36.1	49.4 ± 18.4	0.091^*^
ALP, median (IQR), u/L	222.5 (171.0-584.7)	490.0 (253.0-552.0)	299.0 (231.0-408.7)	343.0 (193.0-503.5)	0.563^δ^
Bilirubin total, median (IQR), mg/dL	2.0 (1.4-4.9)	2.3 (1.2-4.9)	2.6 (1.8-3.6)	2.0 (1.6-4.0)	0.907^δ^
Bilirubin direct, median (IQR), mg/dL	0.3 (0.2-0.3)	0.4 (0.1-0.5)	0.3 (0.2-0.4)	0.3 (0.2-0.4)	0.980^δ^
Hemoglobin, mean ± SD,mg/dl	9.9 ± 0.9	9.7 ± 0.8	9.6 ± 1.3	8.2 ± 1.3	0.061^*^

Abbreviations: ALP, alkaline phosphatase; ALT, alanine transaminase; AST, aspartate aminotransferase; EF: ejection fraction; IQR, interquartile range; LVSF, left ventricular systolic function;PAP, pulmonary arterial pressure; RVSF, right ventricular systolic function; SD, standard deviation * ANOVA test ^#^Fisher exact test ^δ^Kruskal-Wallis test

**Figure 2:**
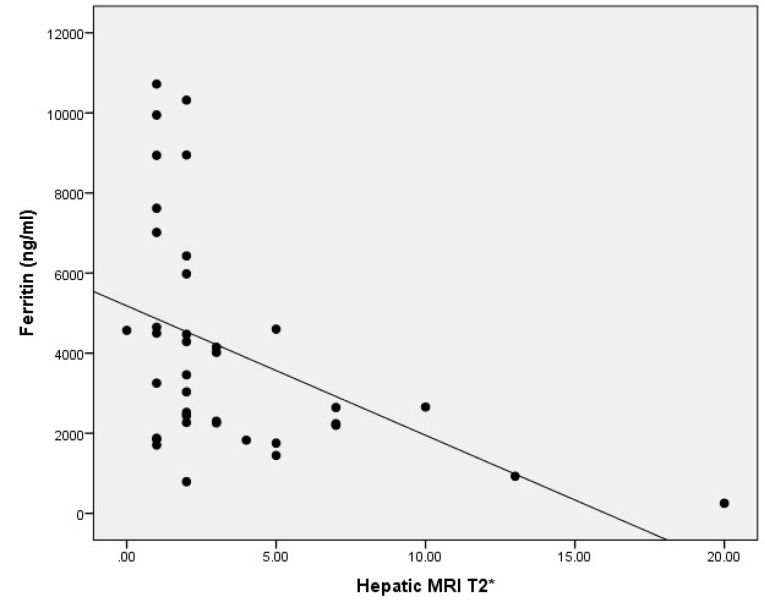
Correlation between hepatic MRI T2^*^ times and serum ferritin levels in β-thalassemia patients (r = -0.52, *P* = 0.001)

Furthermore, liver MRI T2^*^ was negatively associated with serum ferritin level (r = -0.52, *P* = 0.001) (Figure 2).

## Discussion

The present study examined the iron overload status using cardiac and liver MRI T2^*^ in patients with β-thalassemia major. The relationship between T2^*^ results with serum ferritin level, heart function, and liver enzymes activity was also studied. Based on the MRI T2^*^ times, 22% percent and 11% of the patients were suffering from severe iron overload in heart and liver, respectively. Comparison of different categories of heart iron load indicated that only ALT activity was higher in patient with severe iron load than patients with normal iron load. Whilst, serum ferritin and AST levels was significantly different among hepatic iron load categories. There was a significant inverse correlation between serum ferritin level and cardiac iron load and hepatic iron load as well.

Until the development of cardiac and liver MRI techniques in the 2000s, liver biopsy was the principle standard method to assess the body iron burden. However, since liver biopsy is an expensive, unpleasant, and invasive procedure which has inter-observer inconsistency and sampling error because of intra-organ variability in hepatic iron content, its use has been limited. T2^*^ MRI is a noninvasive technique for quantification of iron overload in various organs, particularly the liver and the heart in thalassemia major patients.^16,20^ This method is rapid, simple, and highly reproducible which measures magnetic relaxation properties of each tissue and is inversely associated with intracellular iron deposition.^21^ Therefore, T2^*^ MRI is now considered the gold standard in diagnosis and monitoring of body iron load status and also in the prediction of cardiac failure in patients with thalassemia major.^13,22,23^ Some previous studies reported that cardiac and hepatic iron overload estimated by T2^*^ MRI is correlated with serum ferritin level in thalassemia patients, although the results are inconsistent.^16,20,24,25^ Our finding that showed a significant correlation between liver and heart T2^*^ values and serum ferritin measurements are in keeping with the results of Fischer et al and Voskaridou et al,^25,26^ but in contrast to those indicated by Anderson et al^16^ who could not find a significant correlation. Karakas et al also found that serum ferritin level was significantly correlated with liver T2^*^ values, but not with heart T2^*^ values.^27^ Although, serum ferritin level is the most widely used indirect estimate of iron stores in the body in patients with β-thalassemia, the usefulness of this indicator of body iron is limited by the presence of several clinical conditions including infection, inflammation, and liver disease.^28,29^ So, the inconsistent results may be attributed to coexistence condition that affects the ferritin level and also different sensitivity of MRI T2^*^ for different forms of iron.

Cardiomyopathy due to iron overload is the most common death cause in thalassemia major patients.^30^ Tissue Doppler echocardiography could help to detect wall motion abnormalities, as an early sign of cardiac dysfunction in patients with thalassemia.^31^ In this study, RVSF, LVSF, and PAP was not significantly different among iron load categories based on cardiac and liver T2^*^ values, this is similar to results of Moussavi et al, who could not find any significant correlation between MRI finding and cardiac function.^32^ However, our results are in contrast to those of Anderson et al and Voskaridou et al, who reported correlation of myocardial T2^*^ values with left ventricular ejection fraction (LVEF).^16,26^ The cardiac function was normal in most of our patients (above 77% of patients) even in patients with severe iron overload, this may due to late appearance of echocardiographic abnormalities. Meanwhile, two patients with reduced LVSF had normal cardiac and hepatic T2^*^ MRI. However, lack of the cardiac function differences between iron load categories do not decrease the value of measuring cardiac function in combination of MRI T2^*^ because echocardiography could detect patients with advances disease that need emergency cardiac care.

Liver disease, due to iron overload, is the common complication in thalassemia patients that may manifest by increased ALT and AST level. In the present study, ALT level was significantly higher in patients with severe cardiac iron overload than patients with normal cardiac iron overload. Moreover, AST level was significantly different among patients with different hepatic iron load. Mohammad et al who examined the liver functions in thalassemia patients, found that there was a significant positive correlation between serum ferritin and ALT level.^33^ In a study by Ameli et al, serum ferritin level was significantly greater in patients with ALT level > 40 U/L than patients with ALT level < 40 U/L.^34^ In our study, we also found that serum ferritin level was significantly correlated to ALT and AST level (data not shown). Although, to best our knowledge this is the first study that evaluated the association between liver function and T2^*^ MRI results, the correlation between serum ferritin level and liver enzymes, as well as, the correlation between serum ferritin level and T2^*^ MRI values is shown in other studies.^25-27,33,34^ Therefore, assessment of liver enzymes activity may help to estimate the risk of iron load in thalassemia patients.

This study had some limitations. One of the limitations of this study was that the cross-sectional design which precludes to follow the patients and determine the efficient chelation therapy protocols. Moreover, sample size was relatively small and hepatic iron content was not measured by liver biopsy due to high level of discomfort and funding constraint as well. Another limitation of this study was that we do not consider the diseases can affect the liver enzymes such as hereditary and acquired disease of liver.

## Conclusion

We evaluated the iron load of heart and liver in patients with β-thalassemia major using T2^*^ MRI as a gold standard. Results of cardiac and hepatic T2^*^ MRI showed a significant negative correlation with serum ferritin level. There was not any significant correlation between T2^*^ MRI values and echocardiography results. Moreover, ALT and AST level was significantly different among different cardiac iron load groups and hepatic iron load groups, respectively. Further prospective studies with large sample size are suggested to monitor patients with β-thalassemia major using T2^*^ MRI along with serum ferritin level and liver enzymes.

## Acknowledgements

The authors thank all the patients who participated in the study.

## Competing interest

The authors declared that there is no conflict of interest.

## Ethical approval

The Ethics Committee of Tabriz University of Medical Sciences approved this study (reference number: 9410350), and all patients were asked to sign a written informed consent.

## Funding

None.
